# Defect-Engineered Metal–Organic Frameworks

**DOI:** 10.1002/anie.201411540

**Published:** 2015-06-03

**Authors:** Zhenlan Fang, Bart Bueken, Dirk E De Vos, Roland A Fischer

**Affiliations:** Key Laboratory of Flexible Electronics (KLOFE) & Institute of Advanced Materials (IAM), Jiangsu National Synergetic Innovation Center for Advanced Materials (SICAM)Nanjing Tech University (NanjingTech), 30 South Puzhu Road, Nanjing 211816 (V.R. China); Inorganic Chemistry II—Organometallics & Material Chemistry, Department of Chemistry and BiochemistryRuhr-University Bochum, Universitätsstrasse 150, 44801 Bochum (Germany); Centre for Surface Chemistry and Catalysis, KULeuvenKasteelpark Arenberg 23, 3001 Leuven (Belgien)

**Keywords:** coordination chemistry, defects engineering, heterogeneity, metal–organic frameworks, porous materials

## Abstract

Defect engineering in metal–organic frameworks (MOFs) is an exciting concept for tailoring material properties, which opens up novel opportunities not only in sorption and catalysis, but also in controlling more challenging physical characteristics such as band gap as well as magnetic and electrical/conductive properties. It is challenging to structurally characterize the inherent or intentionally created defects of various types, and there have so far been few efforts to comprehensively discuss these issues. Based on selected reports spanning the last decades, this Review closes that gap by providing both a concise overview of defects in MOFs, or more broadly coordination network compounds (CNCs), including their classification and characterization, together with the (potential) applications of defective CNCs/MOFs. Moreover, we will highlight important aspects of “defect-engineering” concepts applied for CNCs, also in comparison with relevant solid materials such as zeolites or COFs. Finally, we discuss the future potential of defect-engineered CNCs.

## 1. Introduction

In nature, “ideal crystals”, with an infinite periodic repetition or ordering of identical groups of atoms in space do not exist. The structure of “real crystals” always deviates from that perfect arrangement and contains a considerable density of structural irregularities or defects.[1] Crystal irregularities could stem from compositional inhomogeneities, and this concept is often used interchangeably with the term “defects”. In fact, heterogeneity, structural disorders, and defects of various nature and length scales are key attributes of solid-state materials and strongly affect their physical and chemical properties. In some cases, it is desirable to have crystals as perfect as possible (e.g. for optoelectronics).[2] However, defects do not necessarily have adverse effects. Hence, many important material properties rely as much on characteristic imperfections as on the overall “perfect” nature of the host lattice. For example, the electronic properties (i.e. electrical conductivity) of important materials such as silicon or Group III/V compound semiconductors are entirely due to trace amounts of chemical impurities and defects.[2], [3] Many important classes of materials exhibit some form of structural disorder which gives rise to often counterintuitive and useful material properties and functions, such as spin frustration in cooperative paramagnets,[4] thermoelectric,[5] and polar nanodomain formation in relaxor ferroelectrics.[6] Surface defects and interfacial structures of nanocomposites (e.g. metal/support interactions) commonly serve as active sites in heterogeneous catalysis for adsorption and reactive transformations.[7]–[9]

In all these cases, judicious control over the defect structure and associated heterogeneity, that is, “defect engineering”, is of paramount importance to manipulate crystal quality and thus the specific properties desired in a material. A comprehensive analysis of any “real” material structure and performance also requires the identification of the existing defect types, their densities, and distribution, as well as the roles they play in affecting the materials behavior. Being essentially solid-state materials, it is immediately conceivable that metal–organic frameworks (MOFs) should also carry various kinds of defects and may feature structural complexity as a result of heterogeneity.[10]–[12] In this Review, however, we would like to introduce the more fundamental term coordination network compounds (CNCs) for the class of materials that is discussed. Both MOFs and CNCs are based on Werner-type coordination chemistry, where metal ions and/or clusters of metal ions are linked together by oligotopic organic molecules to yield extended network structures. MOFs are essentially a subset of crystalline, (potentially) porous CNCs (*cp*-CNCs). Nevertheless, we also define dense CNCs (*d*-CNCs), which are not porous, amorphous CNCs (*a*-CNCs), as well as flexible CNCs (*f*-CNCs) and rigid CNCs (*r*-CNCs). Depending on the particular structural features of CNCs, certain variations such as *cf*-CNCs, *cr*-CNCs, and *cd*-CNCs are important as well.

Investigations of the (intrinsic) defect structure and the intentional design of imperfections and structural heterogeneity, however, have so far not attracted much attention in MOF, or more broadly CNC, materials research. The intensive activities in the MOF field over the past years have mainly been dedicated to reticular synthesis, new topologies and structures, and potential applications in gas storage/separation,[13] sensing,[14] drug delivery,[15] and catalysis,[13], [16] with little explicit focus on defects. Only recently have the external surface as well as internal imperfections, for example, Schottky-type point defects (such as linker and/or metal node vacancies) been characterized for several “canonical” MOFs.[17]–[23]

The agglomeration or correlation (clustering) of point defects could form (functionalized) mesopores, which in turn could help in overcoming diffusion limitations.[19], [23]–[27] In addition, the modulation of the electronic situation together with the proximate coordination space at so-called coordinatively unsaturated sites of some MOFs, such as HKUST-1 and UiO-66, has been shown to strongly affect their reactive properties.[21], [24], [28] It is the intention of this Review to present the emerging research field of defect-engineered MOFs and CNCs in a systematic fashion. We will discuss different types of defects, structural complexity, and heterogeneity as well as the importance for further development of the materials chemistry (and physics) of such molecular network materials. Naturally, the issue of defect-engineered MOFs, in particular, is likely to attract attention; however, the principles are very general and also other CNCs and molecular network materials in general show interesting properties connected with their defective structure. Of course, in view of the huge current interest in MOFs, the Review will mainly deal with this subclass of CNC materials.

## 2. Definition and Classification of Defects

Various structural disorders and heterogeneities that break the periodic arrangement of atoms have recently been reviewed for CNCs.[29]–[31] Goodwin and co-workers defined statically,[32]–[34] topologically, and dynamically[35]–[41] disordered CNCs[29], [42] on the basis of the type of building blocks, their connectivity pattern and periodicity, as well as atom/unit dynamics. Diverse strategies have been outlined for varying the building blocks within the lattice or the guests inside the pores to introduce heterogeneity without losing long-range order. Furthermore, the integration of distinct types of organic linkers or a combination of homologous linkers bearing different chemical functions, as well as combining various metal-containing secondary building units (SBUs) or more than one kind of metal ion with the same topological role within a single MOF structure were discussed as attractive approaches to adjust the properties of materials.[30], [31] In the latter cases, such manipulations of the parent frameworks often occur in a nonperiodic fashion. Similarly, defects could be essentially considered as a specific form of heterogeneity, implying the nonperiodic removal of some structural elements.

**Figure 1:**
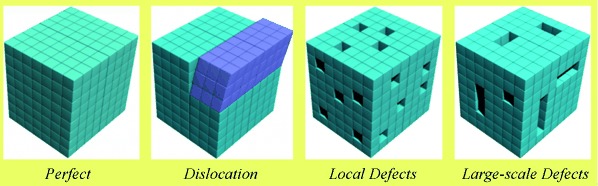
The definition of defects: the missing and incorrectly located atoms generate vacancies and dislocations in materials.

We will restrict our discussion on defects exclusively to *c*-CNCs with much focus on *cp*-CNCs (i.e. MOFs), dictated by the exceptional and continuous interest in this subclass of CNCs. We define defects in CNCs/MOFs as “sites that **locally** break the regular periodic arrangement of atoms or ions of the **static** crystalline parent framework because of missing or dislocated atoms or ions” (Figure 1). In this sense, thermal motion of the constituent atoms or linker rotation is not considered as a defect. Furthermore, an alteration of the parent framework that occurs periodically or homogeneously, for example, the quantitative removal of all solvent molecules coordinated to metal sites simply leads to the formation of a daughter framework featuring coordinatively unsaturated sites rather than leading to a defective form of the parent structure.

In accordance with their dimensions, all structural irregularities in solid materials are conveniently considered under four main divisions: point defects (e.g. vacancies), line defects (e.g. dislocations), planar defects (e.g. boundaries and stacking faults), and micro- and mesoscale volume defects (e.g. inclusions and voids). Additionally, macropores, cracks, and foreign inclusions that are introduced during production and processing of the solids may also be considered here as macroscale volume defects. These classifications could also be adopted for CNCs. According to their location, defects can be designated as external or surface defects[18], [43]–[45] and internal defects.[19]–[24], [27], [46] The latter could originate from partially missing metal nodes or linkers (either the entire molecule or selected functions when fragmented linkers are integrated) and locally break the framework regularity.[19], [20], [23], [46], [47] Such vacancy defects could be considered analogues of Schottky or Frenkel defects in classical solid materials (that is, the local absence of atoms/ions based on the “ideal” crystal structure accompanied by removal of oppositely charged ions or creation of an interstitial defect at the new location, respectively). In the case of defective CNCs/MOFs, charge compensation, if required, is realized through either reduction/oxidation of framework components[24], [46]–[48] or inclusion/removal of respective counterions,[49] either during synthesis or postsynthetically. The formation of linker vacancies may further cause the appearance of modified coordinatively unsaturated metal sites (mCUS)[24], [27], [28], [48] or additional CUSs[21], [46], [49]–[51] that can greatly differ from the regular intrinsic CUSs. Such mCUS could, thus, also be defined as point defects.

Taking into account the distribution, size, and the state of the interaction/correlation of defects in the framework, they can be subsequently divided into two groups: a) local defects, that is, point or isolated defects, and b) large-scale defects (e.g. mesopores; Figure 1). Two distinct situations might arise depending mainly on the concentration of defects within a given structure and the effective size of the defect field. At low defect concentrations and small defect fields, a random distribution of isolated or point defects can be found. On the other hand, high concentrations of defects and/or large fields of defects can result in the formation of correlated or large-scale defects through clustering of point defects. Correlation here means that the probability of forming a defect at a certain location in the crystal lattice depends on the presence of defects in the vicinity of this location. In essence, such large-scale defects are always of higher dimensions. Consequently, correlated linker/node vacancies can generate mesopores that:

- might greatly affect mass-transport pathways (important in sorption and catalysis),
- could reduce network rigidity and density,
- bring out unique electronic, magnetic, and optical functionalities and anomalous mechanical properties (e.g. negative thermal expansion, pressure-induced softening, and crystalline–amorphous switching)
- may bring benefits to realize complex catalytically active sites, for example, rearranged CUSs that can operate in a cooperative manner,[29], [46], [48] for targeted catalytic reactions.

All the CNCs/MOFs reported so far which fall into our definitions of defects and defect engineering are summarized in Table 1, and some of these examples will be specifically discussed below.

**Table 1:** Summary on defective CNCs/MOFs.[a]

Compound	Type of defect(s)	Characterization method(s)	Remarks	Ref.
HKUST-1	plane dislocations with free COOH groups, fractures propagating in the crystal interior	CFM (3D imaging)	activity in Brønsted acid catalyzed reactions (self-condensation of furfuryl alcohol)	[17]
HKUST-1	dislocation growth spirals	high-resolution AFM; atomic-scale representation by modeling	growth spirals at {111} facets; hindered diffusion across dislocation core	[44], [52], [56]
HKUST-1	missing carboxylates on the external surfaces	exposure to CO, CO_2_ and N_2_ monitored by FTIR	dicarbonyl complex on “defective” sites; multiple N_2_ adsorbed at defects	[57]
HKUST-1	temporary defects as Brønsted sites	DFT calculations (periodic model)	high catalytic activity and selectivity in Knoevenagel reactions	[58]
HKUST-1 SURMOF	linker vacancies, extra (partially reduced) mCUSs (ca. 4 %)	In situ UHV-FTIR (CO probe), XPS, DFT calculations	increased reactivity towards CO	[47]
HKUST-1 with fragmented linkers (PyDC, etc.)	modified (extra) mCUSs, partial metal node reduction, linker vacancies	UHV-FTIR (CO probe), TGA-DSC, UV/Vis, NMR, EPR, XPS, magnetic susceptibility, N_2_ adsorption (BET), DFT, EXAFS	modulated band gap and magnetism; enhanced catalytic activity and selectivity in hydroxylation of toluene; altered porosity	[24], [28]
HKUST-1 and NU-125 with fragmented linkers	missing paddlewheel clusters (ca. 8 %), linker vacancies	^1^H and ^19^F NMR spectroscopy, N_2_ adsorption (BET)	altered porosity, H_2_ and CH_4_ uptake_;_ pore functionalization	[59]
Ru analogues of HKUST-1 with fragmented linkers (PyDC)	modified (extra) mCUSs, partial metal node reduction, linker vacancies	TGA, HPLC, ^1^H NMR, XPS, N_2_ adsorption (BET), in situ UHV-FTIRS (CO/CO_2_ probe)	enhanced adsorbate uptake (N_2_, H_2_, CO, C_x_H_y_) and catalytic activity, e.g. in alkene hydrogenation, CO_2_ to CO reduction	[48]
PCN-125 (linker-fragmentation)	modified (extra) mCUSs, linker vacancies	NMR, N_2_ adsorption (BET)	mesopore formation; increase in CO_2_ uptake and heats of adsorption	[27]
MOF-5	surface defects, cracks	CFM (3D imaging), SEM	defects localized in a 10 μm shell	[17]
MOF-5 (microwave heating)	surface defects, grooves	SEM	defects developed on increase in synthesis time (linker dissolution)	[18]
MOF-5 (various synthetic conditions)	square or rhombus terraces with steps parallel to ${ \char60 }$ 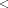 100> or ${ \char60 }$ 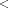 110> direction, respectively	SEM, in situ AFM	crystal growth mechanism at atomistic level is a function of solution Zn^2+^/H_2_BDC ratio	[60]
MOF-5	mesoporous voids	positron annihilation lifetime spectroscopy (PALS)		[61]
MOF-5 and IRMOF-8	mesopores, ZnO species, lattice interpenetration	small-angle X-ray scattering, SEM, N_2_ adsorption (BET)	enhanced H_2_ uptake and storage	[25], [26], [62]
MOF-5 and IRMOF-8	M-OH species	N_2_ adsorption (BET), ^1^H NMR, in situ DRIFTS	alkylation of aromatic compounds with *tert*-butyl chloride, pore shape selectivity	[63]
MOF-5 and IRMOF-3 derivatives (fast precipitation and linker fragmentation)	linker vacancies, M-OH sites and/or included ZnO particles	(MAS)-NMR, TGA, DRIFTS, FTIR (pyridine probe), N_2_ adsorption (BET)	introduction of M-OH Brønsted sites; enhanced catalytic activity e.g. in Knoevenagel condensations and aromatic alkylations	[20], [64]
MOF-5(*Oh*) variant (doping with H_3_BTB)	free dangling COOH groups of H_3_BTB within pores	^1^H NMR, TEM, N_2_ adsorption (BET)	morphology modulation; anchoring of Pd ions on COOH defects	[65]
MOF-5 (DBA as linker fragment)	correlated linker and metal vacancies; sponge- and pomegranate-like crystals	SEM, TEM, N_2_ adsorption (BET), molecular dye inclusion, CFM, small-angle scattering using synchrotron X-ray	enhanced CO_2_ uptake in meso/macropores	[66]
MOF-5 (thermally stressed)	point defects	Green-Kubo method, molecular dynamics simulations	thermal conductivity depends on phonon scattering on defects	[67], [68]
MOF-5 (thermal annealing)	linker fragments by thermal decomposition of BDC	TGA-MS, FTIR, Raman, XPS, N_2_ adsorption (BET)	enhanced CO_2_ uptake and chemical stability	[69]
Zn_4_O(PyC)_3_	ordered linker and metal vacancies upon hydrolysis	single-crystal XRD, PXRD, ^1^H NMR, N_2_ adsorption (BET), inclusion dyes	increased pore size, linker and metal ion exchange on the respective formed vacancies	[55]
UiO-66(Zr)	inherent linker vacancies	TGA-MS, single crystal X-ray diffraction	1–3 of 12 linkers per cluster missing	[64], [70]
UiO-66(Zr) (variations of BDC:Zr^4+^ ratio and synthesis temperature)	linker vacancies	TGA-DSC, NMR, Raman spectroscopy, N_2_ adsorption (BET)	thermal stability dependent on defect concentration; increasing amount of defects with extent of washing	[22]
UiO-66(Zr) derivatives (with BDC-X; X=H, NH_2_, CH_3_, OCH_3_, F, Cl, Br, NO_2_)	linker vacancies, CUSs	first-principles kinetic and molecular modeling calculations	increased catalytic activity in the ene-type cyclization of citronellal	[21]
UiO-66(Zr) (CH_3_COOH linker fragments)	linker vacancies	high-resolution neutron diffraction, neutron inelastic scattering, N_2_ adsorption (BET), dispersion-corrected DFT	increase of pore volume and CO_2_ uptake	[19]
UiO-66(Zr) (TFA linker fragments and HCl)	linker vacancies, mCUSs	TGA, ^19^F NMR, FTIR (CD_3_CN probe), N_2_ adsorption (BET), periodic DFT, molecular dynamics, nudged elastic band theory, free energy diagrams	increased catalytic activity, e.g. in Meerwein–Ponndorf–Verley reduction of 4-*tert*-butylcyclohexanone	[46], [51]
UiO-66(Hf) (formic acid linker fragments)	linker and metal cluster vacancies, correlated defect nanoregions	high-resolution electron microscopy, diffuse scattering, X-ray PDF, TEM, quantum chemical calculations	altered porosity, altered mechanical properties	[23]
UiO-66(Zr)	linker vacancies, M-OH sites	grand canonical Monte Carlo simulations, simulation of water and CO_2_ isotherms	defect-induced hydrophilicity	[71]
Zr squarate (synthesized with modulators)	linkers replaced by monocarboxylate fragments	N_2_ (BET) and H_2_ adsorption, FTIR, TGA, ^1^H NMR	size-selective gas uptake, potential for purification of small gases such as H_2_ or He	[72]
MOF-801-P	linker vacancies	neutron diffraction, N_2_ adsorption (BET)	defect-induced hydrophilicity	[73]
MIL-140A—MIL-140D	mCUSs (<10 % of Zr atoms)	FTIR (CD_3_CN and pyridine probes)	inherent Lewis-acidic defect sites	[53]
ZIF-8, ZIF-9	various defect sites on surface (dangling low-coordinated Zn^2+^, NH groups, N^−^ and OH groups)	FTIR (CO probe), DFT (clusters and periodic models)	catalyst for transesterification of vegetable oils with simple aliphatic alcohols; catalyst for Knoevenagel reaction	[74], [75]
MIL-47	linker vacancies, partial linker decoordination	theoretical calculations	catalytic activity in cyclohexene oxidation with *tert-*butylhydroperoxide	[76], [77]
MIL-53/47 ([(AlOH)_1-*x*_(VO)_*x*_L], L=BDC; NDC)	modified metal nodes	FTIR, EPR, MAS-NMR, N_2_ adsorption (BET) and CO_2_ sorption, in situ synchrotron PXRD upon CO_2_ sorption	modified breathing behavior	[78]
MIL-53(Ga)	internal free COOH defects	CFM (3D imaging)	activity in Brønsted acid catalyzed reactions (self-condensation of furfuryl alcohol)	[17]
MIL-100(Fe) (acid treated)	additional Fe^3+^-mCUSs	N_2_ adsorption (BET), FTIR (CO probe)	enhanced catalytic activity for, Diels–Alder reactions	[49]
CAU-7 (Bi-BTB)	Lewis acidic defect CUSs	FTIR (CD_3_CN probe)	weak Lewis acid catalyst for selective hydroxymethylation of 2-methylfuran	[79]
NOTT-202	slits formed by defective, partially occupied interpenetrated second network	single-crystal XRD, grand canonical Monte Carlo simulations, N_2_, CH_4_, O_2_, Ar, and H_2_ sorption	temperature-dependent adsorption/desorption hysteresis; selective response to CO_2_	[36]
MOF-505	metal vacancies (ca. 0.57 %)	XPS, EXAFS, magnetic measurements, DFT	long-range ferromagnetic coupling	[80]
[Ba_2_(BTC)(NO_3_)] (thermally treated)	mCUSs, bridging O^2−^ sites formed by selective nitrate decomposition	TGA-MS, SEM, EPR, DFT	base catalytic activity in Michael addition reaction	[50]
Fe^III^_4_[Fe^II^(CN)_6_]_3_⋅*x* H_2_O; (NH_4_)_0.7_Fe^III^_1.10_[Fe^II^(CN)_6_]	Fe(CN)_6_^4−^ vacancies	TGA-MS,	H_2_O and Cs^+^ adsorption	[81]
[Cu_5_(BPP)_8_(SO_4_)_4_ (C_2_H_5_OH)(H_2_O)_5_](SO_4_)	surface cracks (ca. 1.5–4 μm wide)	AFM	cracks are formed upon desolvation and run mainly orthogonal to {010}	[45]
[Cu(BPP)_3_Cl_2_]_3_⋅2 H_2_O	screw dislocations at {001} faces	in situ AFM	screw disclocations starting from growth hillocks	[82]
Ru^II^bpy-templated ZnBTB (RWLC-2)	defect voids	single-crystal XRD, steady-state and time-resolved emission	Ru^II^bpy encapsulated in defects with quencher reduces emission lifetime	[83]

[a] BDC: 1,4-benzenedicarboxylate, BPP: 1,3-bis(4-pyridyl)propane, bpy: 2,2′-bipyridine, BTC: 1,3,5-benzenetricarboxylate, BTB: 1,3,5-benzenetrisbenzoate, DBA: 4-(dodecyloxy)benzoic acid, NDC: 2,6-naphthalenedicarboxylate, PyC: 4-pyrazole carboxylate, PyDc: 2,5-pyridinedicarboxylate, TFA: trifluoroacetic acid.

## 3. Formation of Defects

### 3.1. Inherent Defects

**Figure 2:**
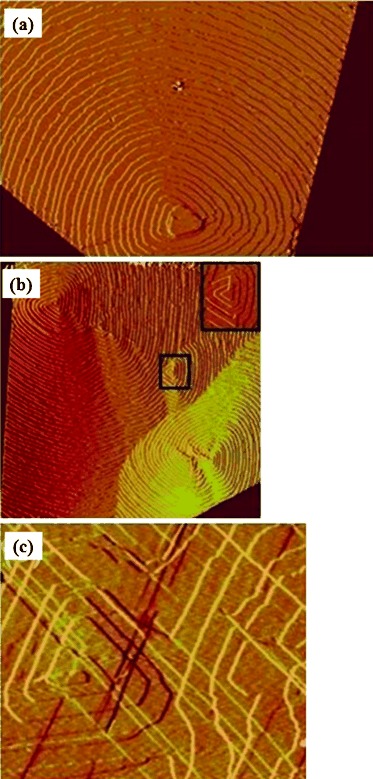
AFM amplitude images of {111} facets of HKUST-1, showing a) a double-growth spiral, b) merging single and multiple growth spirals as well as c) growth spirals overlaid with fractures primarily in the ${ \char60 }$
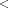
110> directions.[44]

Inherent defects are formed during crystal growth as a result of stacking faults or dislocations and can occur without targeted engineering. Inherent means that no other manipulations were performed during synthesis besides mixing the normal building blocks of the parent framework under regular synthetic conditions. CNC/MOFs are prone to the formation of inherent defects arising either from misconnections or dislocations during crystallization[44] or from postcrystallization cleavage.[45] An example of such misconnections are screw dislocations, which are evidenced by growth spirals on the crystal surface of HKUST-1 ([Cu_3_(BTC)_2_], BTC=benzene-1,3,5-tricarboxylate) and MOF-5, which are observed by atomic force microscopy (AFM; Figure 2).[44], [52]

Inherent internal defects are also found in several MOFs, for example, in the form of free, pendant carboxylate groups or missing linkers.[20], [21], [23], [53], [54] Their formation is often affected by the synthetic conditions. In fact, increasing the reaction temperature and starting linker/metal ratio may considerably decrease the concentration of linker vacancies.[55] The precipitation rate is also an important factor and its increase could favor vacancy formation. It is clear that (very) fast crystal growth may not ensure enough time for the MOF building blocks to quantitatively adhere to the growing crystal lattice at the right place, and defects cannot be corrected if the coordination bonding is insufficiently reversible. Indeed, the fast crystallization of IRMOF-3 and MOF-5 leads to the formation of terephthalate (BDC) “holes” with simultaneous decoration of the internal surface with Zn-OH sites, as revealed by FTIR analysis.[20] This is a simple and convenient method to intentionally generate new catalysts with an acidity and hydrophilicity different from those of the parent MOFs. Some authors have investigated the formation of inherent defects during the growth of membranes and thin films of MOFs; they emphasized the importance of the quality of the self-assembled monolayer substrates (SAMs) used, as their contamination or defective structure can result in a considerably defective surface-mounted MOFs (SURMOFs).[84], [85] Other relevant examples will be mentioned below.

### 3.2. Defect Engineering

#### 3.2.1. Defects Formed during De Novo Synthesis

The great modularity of CNCs/MOFs enables the intentional introduction of various types of defects while retaining the overall structural integrity.

##### 3.2.1.1. Solid-Solution Approach: Coassembly of Mixtures of Ligands or Ligand Fragments

**Figure 3:**
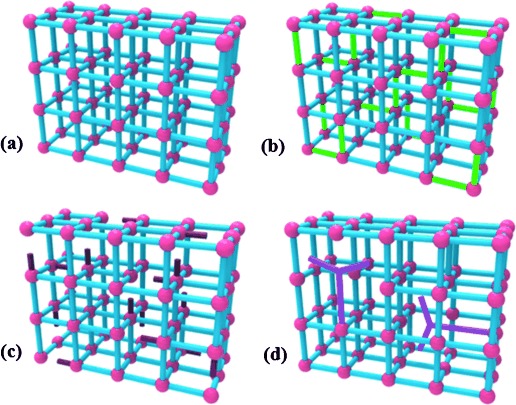
Illustrations of a) the perfect MOFs, b) the heterogeneous MOFs with an isostructural mixed linker (IML), the defective MOFs with a heterostructural mixed linker (HML) by c) the large mixed linker (LML) and the d) truncated mixed linker (TML) approaches for framework functionalization.[86]

The solid-solution approach, which involves mixing two or more organic linkers directly in a reaction mixture, is a straightforward procedure, which has already been successfully used for MOFs. The type of resulting mixed-linker MOF depends on the nature of the incorporated linkers, while the framework topology is typically preserved. For example, using isostructural mixed linkers (IMLs) with different secondary functionalities (e.g. side groups) but identical linker topology and ligator functionality leads to heterogeneous MOFs (Figure 3).[86]–[89] Alternatively, the heterostructural mixed linker (HML) strategy uses organic components with different linker topology or structure. Crystalline CNCs with heterostructural linkers may require an ordered distribution of the topologically different linkers in the framework. However, other situations are possible. The kinetics of framework crystallization determine the role of the doping heterostructural linker which may act as a capping agent, thereby directing the crystallite morphology and surface chemistry,[90], [91] or can functionalize the framework interior.[20], [27], [65], [86] Thus, depending on the connectivity, size, and secondary functionality of the added linker, the HML strategy can be used in two ways: a) the large mixed linker (LML) approach which utilizes larger linkers that feature a higher connectivity compared to the parent linker;[65] b) the truncated mixed linker (TML) approach (also called ligand-fragment coassembly), where molecules with lower connectivity, that is, linker fragments are employed. The TML concept is strongly related to the modulation approach, whereby a monocarboxylic acid is added during synthesis to influence crystal growth and morphology.

**Figure 4:**
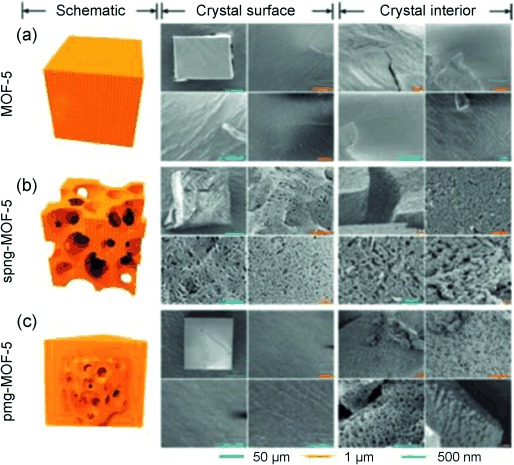
Pore structures of MOF-5, spng-MOF-5, and pmg-MOF-5 revealed by SEM observation of their crystal surface and interior (scale bars, green 50 μm, red 1 μm, blue 500 nm).[66]

The LML strategy is scarcely used, as it often yields only physical mixtures of different phases. The only example comes from the Matzger research group, who prepared defective MOF-5 by doping with 1,3,5-benzenetrisbenzoic acid (H_3_BTB),[65] which triggered a change in the crystal morphology from cubic to octahedral. In contrast, the TML approach is more often applied, for example for MOF-5 and Cu_2_ paddlewheel based MOFs (HKUST-1, NOTT-101,[27] and NU-125).[59] Choi et al. replaced large amounts of 1,4-benzenedicarboxylic acid (H_2_BDC) in the synthesis of MOF-5 by 4-(dodecyloxy)benzoic acid (DBA), which resulted in significant textural changes of the crystals.[66] Curiously, when 50 % of the H_2_BDC was replaced, a spongelike morphology was obtained with large meso- and macropores that permeate throughout the entire crystal (Figure 4). However, when only 30 % of the H_2_BDC was substituted, a pomegranate core–shell-type structure was formed, where the central core features the same sponge morphology, but is enclosed by a shell of intact MOF-5. During crystal growth, DBA seems to coordinate to the growing crystallites and locally impedes crystal growth because of the long alkyl side chains, which eventually leads to the formation of meso- and macropores. Hence, DBA promotes the formation of correlated defects. DBA is removed postsynthetically during the washing steps, thereby yielding highly accessible mesopores.[66] DBA serves a dual purpose as a truncated linker and also as space-filling agent that directs pore formation. Hierarchical dual porous structures with large internal surfaces (mesopores) have also been observed for HKUST-1.[92]

Another well-studied MOF for which TML synthesis has proven its merit is the Zr-terephthalate UiO-66. In fact, the use of monocarboxylate modulators (e.g. benzoic, acetic, or trifluoroacetic acid, TFA) in the synthesis of UiO-66 facilitates control not only of the crystallite size[93] but also of the formation of defects. Thus, the modulator here acts as a truncated linker. Importantly, the concentration of defects in the resulting MOFs relies on the modulator concentration in the synthesis mixture, thus allowing a controlled increase of the solid porosity.[24] Furthermore, Vermoortele et al. showed how the incorporation of TFA opens up more Lewis-acidic mCUSs on the Zr_6_ clusters.[46] The TFA could be removed from the already defective framework by thermal treatment, thereby resulting in a further increase in the number of Zr-CUSs.

##### 3.2.1.2. Metal Node Vacancies

Metal ion/node vacancies can occur in a similar way to, or possibly even simultaneously with, linker vacancies. However, reports of such defects are scarce. One example concerns copper vacancies in some MOFs featuring paddlewheel clusters.[80] Similarly, when using isophthalates as linker fragments in the synthesis of HKUST-1, up to 8 % of the Cu dimers were reported missing in the defective material.[59] Moreover, Cliffe et al. showed that in UiO-66(Hf), besides linker vacancies, entire Hf_6_ clusters can be missing.[23]

#### 3.2.2. Defect Formation by Postsynthetic Treatment

**Figure 5:**
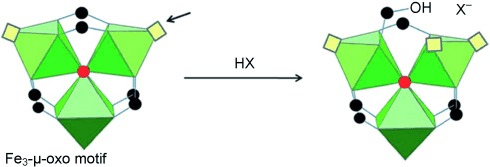
Proposed acid-activation mechanism of the Fe_3_O cluster by a Brønsted acid (HX). A new mCUS is opened up on Fe (yellow squares).[49]

Acid/base postsynthetic treatment has been demonstrated to be an efficient strategy for introducing defects into preformed MOFs. For example, when [Fe_3_O(BTC)_6_(OH)(H_2_O)_2_] (MIL-100(Fe)) was treated with TFA or HClO_4_, reprotonation of one of the BTC linkers at the Fe trimers occurred with the concomitant formation of additional Lewis-acidic CUSs (Figure 5). However, the loss of one negative charge of the linker necessitates the incorporation of counteranions in the pores of acid-treated MIL-100(Fe); these could decrease the porosity of the defective material.[49]

Defect sites can also be formed during solvent exchange and subsequent material evacuation/activation. For example, Shearer et al. observed an increase in the concentration of linker vacancies in UiO-66 with increased washing, possibly through hydrolysis.[22] Similarly, the immersion of [Zn_4_O(PyC)_3_] (PyC=4-pyrazolecarboxylate) in water leads to the formation of vacancies. Interestingly, over the course of 24 h, up to half of the linkers and 25 % of the Zn ions are leached from the framework, thereby resulting in a new lower symmetry phase with ordered or correlated metal and linker vacancies.[55] A recent report revealed that thermal treatment of MOF-5 at temperatures below the decomposition point of the framework but far above the conventional evacuation temperatures induces the in situ decarboxylation of BDC, effectively generating linker fragments postsynthetically.[50], [69]

## 4. Defect Engineering in CNCs/MOFs versus Related Inorganic and Organic Materials

CNCs are molecular network materials. They can be regarded as hybrid materials with properties between those of inorganic networks, such as zeolites, and purely organic frameworks, such as activated carbons or porous organic polymers. While conventional (porous) inorganic solids consist mainly of Al, Si, O, and P, the almost infinite combinations of metal ions and organic linkers results in CNCs/MOFs featuring a higher degree of chemical and structural diversity. The relatively low bond energies of coordination bonds (15–50 kcal mol^−1^) lead to a certain lability and in some cases kinetic reversibility of the coordination bonds.[94] Therefore, it is expected that defect engineering in CNCs/MOFs allows more degrees of freedom for rational design and as a consequence a much better physicochemical understanding of the defects, their formation, and implications could be gained. For zeolites, the most common “defect” is the substitution of Si^4+^ by Al^3+^.[95], [96] As a consequence of charge neutrality, such isomorphous substitution is accompanied by the formation of hydroxy groups, which is the main reason for the existence of Brønsted acidity in zeolites. This is in analogy to the mixed-metal CNC/MOF solid solutions which display similar phenomena. Local metal exchange can be described as substitutional point defects or impurities. On the other hand, in inorganic aluminosilicates, selective removal of Al atoms (dealumination) or Si atoms (desilication) could be achieved by steaming or acid/base leaching.[95]–[97] Likewise, a postsynthetic treatment with H_2_O_2_ is commonly employed to remove titanium.[97] Such network modifications, as in certain cases of isomorphous metal substitution, give rise to additional internal OH groups. The removal of even more significant fractions of framework cations leads to the formation of a mesoporous matrix throughout the zeolite crystal, thereby increasing the pore volume and surface area of the zeolite. In a way, these processes are similar to the formation of linker vacancies in MOFs. A peculiar observation in terms of the formation of these network materials was reported by Meza et al. They showed that surface nucleation and terrace spreading during silicalite synthesis can be switched on/off through careful control over the silicate supersaturation. Insufficient rates of terrace spreading relative to surface nucleation rates lead to the incorporation of defects in the framework, which opens up the possibility to control the defect density and intergrowths through a subtle control of the synthetic conditions.[98] Likewise, fast precipitation and modulation approaches have been used to control the nucleation, growth, and crystal defectiveness of CNCs/MOFs.[20], [23]

Defect engineering in covalent organic frameworks (i.e. COFs, often crystalline and porous solids) and related organic materials is in its infancy. Interestingly, the studies by Ourdjini et al. on self-assembled surface-confined COFs illustrate that defect density crucially depends on the targeted dimensionality. Thus, 2D-ordered COFs always contain topological defects, while lower dimensional chains/ribbons are almost always more or less perfect.[99] Furthermore, in analogy to MOFs, the TML or fragmented-linker concept afforded defect incorporation in COF-102. By co-condensing a trigonal trisboronic acid with the parent tetrahedral monomer, a COF-102 with over 30 % incorporation of the defective monomer was synthesized.[100] Moreover, a fast precipitation synthesis was likewise useful to strongly increase the microporosity of porous organic cages.[101] Liang et al. recently reported on the chemical modification of electrical defects in a prototypical organic semiconductor, regioregular poly(3-hexylthiophene) (P_3_HT).[102] Treatment of P_3_HT, either with LiAlH_4_ or Me_2_SO_4_, resulted in elimination of some of the p/n-type defects, analogous to p/n-type doping. A simultaneous treatment with both compounds further strongly decreased the defect density, thus leading to an improvement in the material performance, especially in terms of stability against photodegradation. Similar defect engineering and defect correction could be envisioned also for COFs when aiming at fine-tuning semiconductor properties.

## 5. Characterization of Defects in CNCs/MOFs

### 5.1. Experimental Studies on Analyzing Defects

The discussion on defective CNCs/MOFs has so far primarily focused on their synthesis and properties. As a consequence of the lack of suitable characterization methods and developed analytical procedures, a molecular-level understanding of the electronic and steric properties at the defective sites is limited. Another major challenge is to establish fundamental correlations between the defects and properties of the resulting defective materials. In this section we will highlight some of the most useful techniques for the physicochemical analysis of defective CNCs/MOFs.

**Figure 6:**
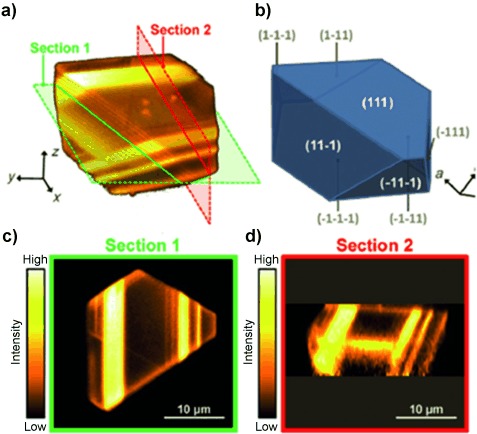
Top: 3D CFM image of an HKUST-1 single crystal obtained after an extended crystallization time.[17] Bright planes represent planes of COOH dislocations. Bottom: 2D sections through the 3D reconstruction.

Long-range defects can be directly imaged using AFM, SEM, and TEM. For example, AFM snapshots can reveal the formation of cracks through desolvation,[45] or AFM can be used to image the formation of growth spirals associated with screw dislocations, as for HKUST-1 (see Figure 2), [Cu(1,3-bis(4-pyridyl)propane)_3_Cl_2_]⋅2 H_2_O,[82] and MOF-5.[60] Furthermore, force modulation microscopy (FMM) is used extensively to image surface defects and compositional changes in composite materials. Confocal fluorescence microscopy (CFM), which has been widely applied in biological imaging, has found application also in the imaging of porous materials to visualize defects that are confined to the crystal interior. For example, it has been used to study the initial catalytic activity of individual zeolite H-ZSM-5 crystals by monitoring the intracrystalline location of fluorescent products obtained by the acid-catalyzed self-condensation of furfuryl alcohol inside such crystals.[103] Ameloot et al. recently used CFM to image defects in HKUST-1 crystals, relying on the same furfuryl alcohol self-condensation, catalyzed by acidic framework defects.[17] 3D CFM images were constructed from a series of 2D micrographs of cross-sections through the crystals. These 3D images help to visualize the penetration of surface defects into the crystal interior. In this way, the main crystallographic directions along which plane dislocations result in free, pendant carboxylic acid groups could be identified (Figure 6). CFM was also a useful tool for imaging postsynthetic defect formation and assessing the catalytic activity in [Ba(BTC)(NO_3_)].[50] Thus, CFM can be used to judge the relative merits of different routes for the preparation of catalysts and to evaluate MOF stability associated with defect formation.

**Figure 7:**
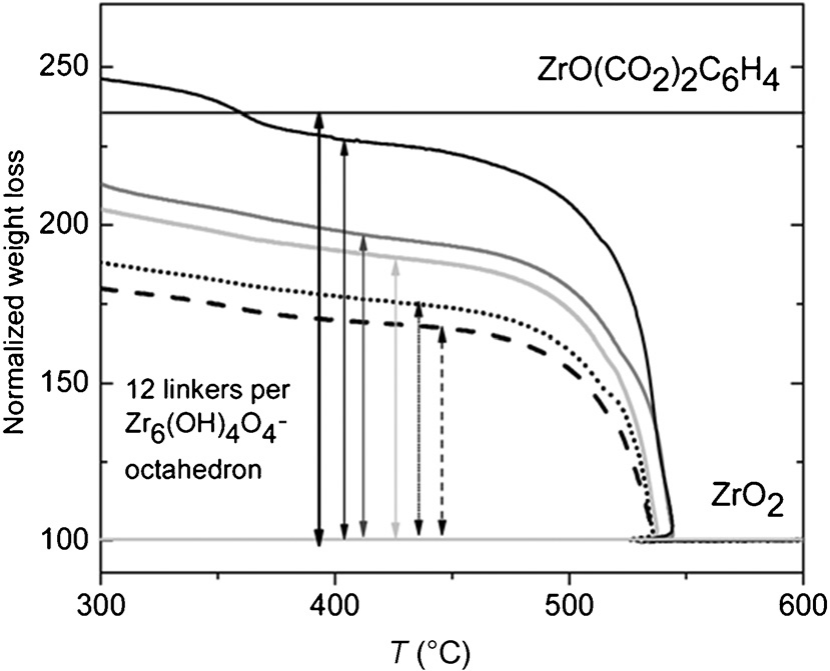
TGA curves of UiO-66 samples from different synthesis batches, indicating the presence of linker vacancies. The theoretical weight loss for a fully coordinated UiO-66 is indicated by the bold vertical arrow between the two horizontal lines.[54]

Thermogravimetric analysis (TGA) is one of the most accessible methods that can provide first-hand information to assess the presence of defects. A representative example of its use is found in the study of UiO-66, built up from [Zr_6_O_4_(OH)_4_] clusters interconnected by a maximum of 12 BDC linkers. On the basis of TGA data it was estimated that about 1 to 3 out of 12 linkers is inherently missing at each cluster (Figure 7).[54] Apart from an estimation of the stoichiometry, TGA, particularly when coupled with MS, provides reliable information on the type and quantity of incorporated guest molecules, which commonly increases for the same structure upon the creation of defects. This was shown, for example, for Prussian Blue structures with lattice defects in the form of missing [Fe^II^(CN)_6_]^4−^ moieties. Solvated water, which fills up such vacancies, strongly favors Cs^+^ adsorption.[81] Finally, several studies reported lower thermal stabilities for defect-rich MOFs in comparison with their “nondefective” analogues.[22]

In certain cases, for example, when mixed or fragmented linkers are used, the introduction of defects might easily be confirmed by routine spectroscopic techniques, for example, FTIR, UV/Vis, diffuse reflectance (DR), or Raman spectroscopy. For example, Lillerud and co-workers found that the Raman spectrum of defective UiO-66 features splitting of several bands associated with carboxylate groups and a concomitant weakening of the fingerprint vibrations below 500 cm^−1^.[22] Furthermore, NMR spectroscopy and HPLC analyses of digested samples are also used to qualitatively and quantitatively assess the inclusion of distinct linkers, while CP-MAS NMR spectroscopy may provide information on the chemical state of the linkers and linker fragments.[20], [46]

Typically, defective derivatives retain the long-range order and topology of the parent framework, but suffer from losses in short-range order because of the presence of randomly located defects. Therefore, their powder X-ray diffraction (PXRD) patterns should provide similar symmetry and lattice information as the patterns of the respective parent frameworks. Thus, although being the most common technique for structure determination/identification, PXRD generally provides little information on defects. However, high-resolution neutron scattering studies can provide direct structural evidence for linker vacancies, as has been recently demonstrated for UiO-66.[19] As the X-ray scattering cross-section of each element is proportional to the square of its atomic number, the XRD pattern of UiO-66 is dominated by the heavy Zr atoms, while being rather insensitive to the lighter elements of the linker molecules. On the other hand, organic linkers and metal centers are equally sensitive to neutron diffraction as a consequence of the similar neutron-scattering cross-sections of Zr, O, C, and D. Consequently, through structural refinement, the refined linker occupancies derived from data recorded at 4 K and 300 K were determined to be 91.7 and 89.0 %, respectively, which corresponds to 1 out of 12 linkers missing, in good agreement with the TGA results. Finally, single-crystal XRD could provide extremely valuable insight into the defect structures of systems available as sufficiently large single crystals (ca. 5–100 μm).[42] For example, the Lillerud group was able to refine the UiO-66 structure and reveal an approximate 73 % occupancy of the BDC linker based on synchrotron measurements (*λ*=0.760 Å) on single UiO-66 crystals.[70]

**Scheme 1:**
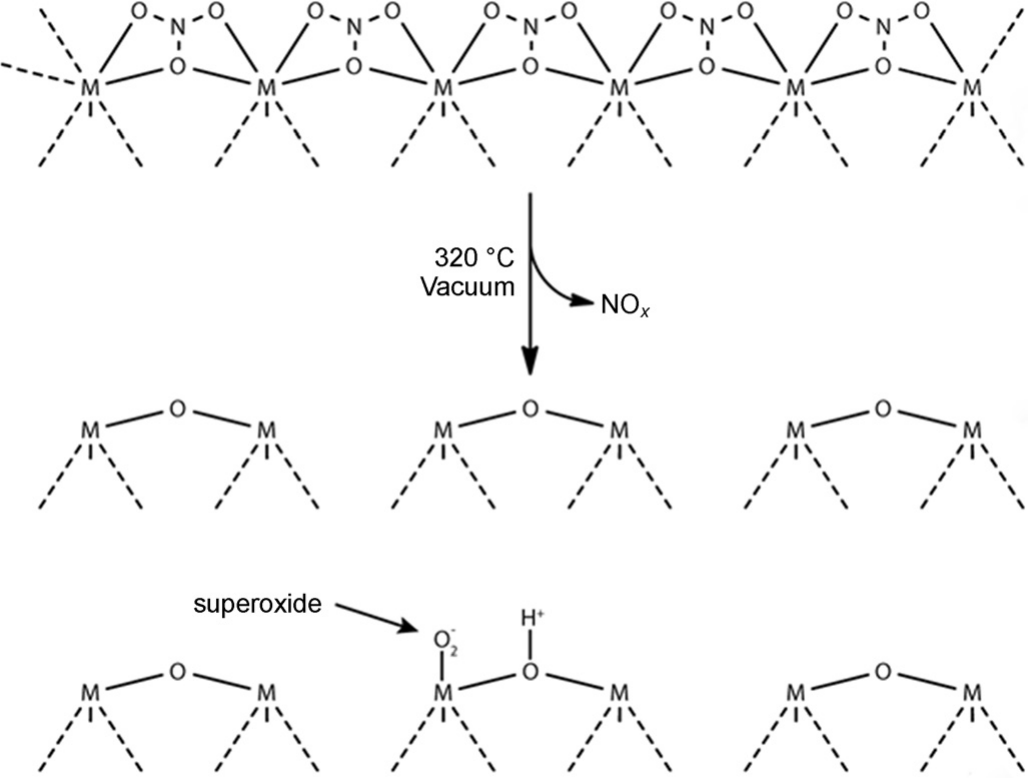
Schematic representation of mCUS and superoxide formation upon activation of [Ba_2_(BTC)(NO_3_)]; M=Ba.[50]

A deeper understanding of the structural and electronic changes at the defect sites requires methods that are more sensitive to local chemical environments. This toolbox includes electron paramagnetic resonance (EPR), extended X-ray absorption fine structure (EXAFS), and X-ray absorption near-edge structure (XANES) analyses. For example, by using EXAFS and XANES measurements, Baiker et al. proved the general decrease of the coordination number of Cu ions upon doping of HKUST-1 with the modified/fragmented linker 2,5-pyridinedicarboxylate (PyDC), thus proving its framework incorporation.[28] Moreover, EXAFS in conjunction with X-ray photoelectron spectroscopy (XPS) and energy-dispersive X-ray spectroscopy (EDX) data enabled Yang and co-workers to confirm the presence of Cu vacancies in MOF-505 and to determine their concentration.[80] Remarkably, the EXAFS spectrum of MOF-505 measured at the Cu K edge lacks the Cu-Cu coordination peak evident in Cu acetate, thus, strongly indicating the presence of Cu vacancies. Valvekens et al. employed EPR measurements to study the low-coordinated Ba sites in their postsynthetically modified defective material [Ba(BTC)(NO_3_)] (Scheme 1).[50] Exposure to molecular oxygen results in the formation of superoxide ions on the Ba sites; the superoxides are readily identified in the resulting EPR spectra. Finally, certain combinations of the aforementioned analyses can shed light on the distribution of defects. For example, when diamagnetic [Al(OH)L]_*n*_ MOFs (L=BDC or NDC) were doped with paramagnetic V^4+^ centers, MAS-NMR and EPR spectroscopy could be used to prove the random distribution of these defects.[78]

One the most powerful methods to characterize the properties of CUSs in MOFs is FTIR spectroscopy with various probe molecules. It has found widespread application in the characterization of the acid-base properties of oxides and porous materials.[104]–[106] Several molecules including CO, CO_2_, and CD_3_CN appear to be the most suitable probes for studies on MOFs. For example, Vimont et al. used CO to assess the Lewis acidity of MIL-100(Cr).[107] Following this successful approach, similar investigations have also been conducted on the parent HKUST-1[108] as well as on defect-engineered MIL-100(Fe).[49] Furthermore, CO chemisorption and thermal desorption monitored by ultrahigh-vacuum (UHV) FTIR[109], [110] has recently been used by Fischer and co-workers to characterize the local environments of mixed-valence Cu^2+/+^ paddlewheel nodes in defective HKUST-1.[24] The data, in conjunction with accurate density functional theory (DFT) calculations afforded insight into the structural and electronic properties of the formed mCUSs. The optimized Cu_2_ paddlewheel structures were consistent with one CO molecule coordinating to a regular Cu^II^_2_(BTC)_4_ unit and two CO molecules coordinating to the defective Cu^II^Cu^I^(BTC)_3_ units with linker vacancies. Analogous experimental and theoretical studies were also conducted on HKUST-1 thin films, where two dominant CO FTIR signals at high and low frequency for the Cu^2+^ and Cu^+^ ions, respectively, were observed, thus indicating the presence of defective units. Thus, high-resolution UHV-FTIR measurements can serve as a quality-control indicator for defects.[47] CD_3_CN was used by several groups to study the number and strength of Lewis-acidic defects in materials such as the Bi-BTB MOF CAU-7[79] or in Zr-based MOFs, for example, MIL-140[53] and the UiO-66 series.[46] While a negligible influence on acid strength was observed in the defect-engineered UiO-66, the amount of Lewis-acidic CUSs on Zr^4+^ increased from 0.72 mmol g^−1^ in the nonmodulated material to 1.1 mmol g^−1^ in the defective MOF when TFA was added to the synthesis mixture. The latter corresponds to two Zr-CUSs per cluster.[46] Finally, pyridine was applied as a probe by Ravon et al. to investigate defects introduced in MOF-5 and IRMOF-3.[20]

The abovementioned methods are particularly helpful to detect and characterize local defects and to probe their chemical and physical environment. However, they do not distinguish between isolated and correlated defects. One indication for defect clustering is the characteristic type IV isotherm for N_2_ adsorption. In fact, by identifying this sorption behavior, several groups could prove that the clustering of numerous local defects results in larger-scale mesoporosity for HKUST-1,[24] UiO-66, and PCN-125.[19], [27], [46] Calculation of the Brunauer–Emmett–Teller (BET) surface area also revealed a relationship between the defect concentration and porosity.[19], [22], [24], [27] However, there is no stringent connection between the change in surface area or pore volume and the employed method of defect engineering. When the concentration of defects is sufficient to create mesopores, small-angle X-ray scattering can also be useful to study the morphology and approximate size of these voids in MOFs.[25], [62]

**Figure 8:**
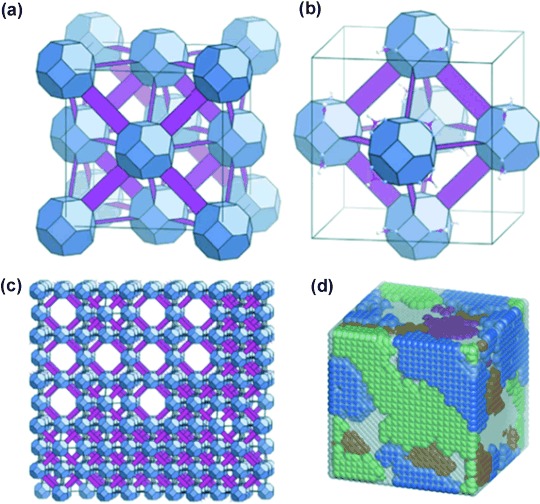
Structural model of defect nanoregions in UiO-66(Hf) deduced from a set of complementary analytical methods. a) Polyhedral representation of a single unit cell of UiO-66. b) Polyhedral representation of a single unit cell of the (ordered) defect structure. c) Defect-rich nanoregions are dispersed throughout a matrix of a defect-free framework. d) Atomistic model of defect nanoregions in UiO-66(Hf) Different colors correspond to different orientations of the defect regions with respect to the bulk UiO-66 (gray).[23]

Ordered defect correlation is different from the random defect clustering that forms mesopores in some materials. Nanoregions with an ordered (correlated) structure of missing nodes have been found in defect-engineered UiO-66(Hf) (Figure 8).[23] In fact, symmetry-forbidden PXRD reflections were observed for such UiO-66 samples and its functionalized analogues.[111], [112] These reflections could be indexed in the same cubic cell by lowering the symmetry from face-centered to primitive; however, their origin remained unclear. Furthermore, these diffuse scattering peaks were revealed to be strongly dependent on the synthetic conditions. Recently, Goodwin and co-workers used a combination of theoretical and experimental techniques to rigorously attribute the presence of the superlattice reflections to correlated defect nanoregions. The authors concluded that primitive nanodomains arise within UiO-66(Hf) through correlated linker and node vacancies. These domains adopt the **reo** topology with eight-connected clusters as opposed to the **fcu** topology of the parent lattice which contains twelve-connected nodes, while retaining an almost similar cell parameter. An atomistic model that accounts successfully for both the sharp Bragg peaks and the broad diffuse scattering component in PXRD pattern was constructed (Figure 8). As a consequence of the simultaneously missing linker and nodes, the rather complex framework composition cannot be deduced by TGA or neutron diffraction alone.[23]

### 5.2. Theoretical Methods To Model Defects in CNCs/MOFs

Molecular modeling is a powerful and essential complementary tool for understanding the nature and structure of defective sites and their distribution through the framework matrix (Figure 8); it is equally important for elucidating the origin of the enhanced or changed adsorption or reactive properties of defect-engineered CNCs/MOFs.

Dislocations and stacking faults create a set of extended microscale crystal defects which can radically alter material properties. However, it remains a major computational challenge to determine the influence of such defects on the atomic level. By applying a newly developed simulation technique, Walker et al. have successfully modeled the structure of screw dislocations in zeolite A.[43] The pore system is perturbed at the location of the screw dislocation, thereby causing local blocking of the pores that connect perpendicular to the channel containing the dislocation line, while the neighboring channels are instead only deformed from a circular to an oval cross-section. The predicted channel structure, and generated local chiral sites, should considerably affect the transport of molecules from the surface to the interior, while retarding transport parallel to the surface. Related studies on HKUST-1 revealed similar results.[56] In this latter case, the modeled screw dislocations run through the Cu_2_ dimers, which are no longer fully coordinated by the BTC linker, but instead by monomeric ions (e.g. -OH); in this way, charge neutrality of the framework is preserved.

**Figure 9:**
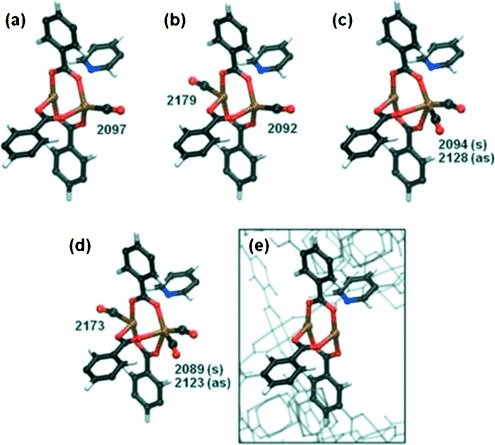
QM/MM-computed binding modes of CO. a–d) QM/MM models of the local mixed-valence defect Cu^II^/Cu^I^(BTC)_3_(PyDC) and energetically feasible binding modes for one to three adsorbed CO molecules (only the QM system is shown for clarity; Cu brown; C black; O red; N blue; H white) together with the computed (scaled) CO stretching normal-mode frequencies (cm^−1^). e) The defect (QM system) embedded in the MM environment.[24]

Quantum mechanical/molecular mechanical (QM/MM) calculations were applied to investigate the local defects at CUSs in HKUST-1 doped with PyDC (Figure 9).[24] The studies confirmed a significant modification of the proximate coordination space and a simultaneous change in the electronic properties of the MOFs upon incorporation of PyDC. In accordance with the experimental studies, the incorporation of PyDC facilitates the formation of mixed-valence Cu^2+^/^+^ paddlewheel nodes compared to the parent framework which contains only a small fraction of inherent Cu^+^ sites (<5 %). Importantly, as a consequence of the generation of additional coordination space around the metal sites, more CO probe molecules could be adsorbed at the mCUSs, with higher calculated binding energies relative to the native CUSs.

Similar calculations were performed on UiO-66 to study its catalytic activity in the cyclization of citronellal. Vermoortele et al.[21] reported that realistic transition states for this reaction could only be found for defect cluster models, that is, lacking at least one linker. Subsequent studies succeeded in controlling the number of defects by incorporation of TFA as a linker fragment.[46] Periodic DFT calculations revealed that the free energy needed to remove TFA from the Zr_6_ cluster equals 24.7 kJ mol^−1^, which indicates that high-temperature activation of such modulated UiO-66 materials results in a further increase in the defect site number, perfectly in line with the experimental data. From the construction of free-energy diagrams, it became apparent that the formation of defects in UiO-66 is not thermodynamically favored. However, this barrier is significantly reduced in the presence of TFA due to entropic effects. Thus, when there is a high concentration of TFA in the synthesis, the formation of linker vacancies becomes very likely despite the higher free energy associated with this defect type.[78] A similar approach was applied to defects generated in [Ba(BTC)(NO_3_)]⋅DMF, where DFT-based simulations were used to calculate proton affinity values for Ba-CUSs.[50] These calculations showed the defect sites on Ba to be more basic than in the native material.

Lastly, grand canonical Monte Carlo simulations were employed by Snurr and co-workers to simulate the water adsorption behavior of defective UiO-66(Zr).[71] By creating a unit cell in which one missing linker was replaced by four hydroxy groups, they simulated that water uptake already starts at 0.1–0.2 *p*/*p*^0^, while for the nondefective UiO-66 this only occurs at 0.8 *p*/*p*^0^ and with a lower saturation loading for the parent MOF. The shape of the measured isotherms more closely fits to simulated isotherms of defective materials, albeit with the adsorption onset at higher *p*/*p*^0^ and lower saturation values, thus indicating that the real samples contain fewer vacancies than the modeled ones. In this case, the rather hydrophobic UiO-66 is thus imbued with hydrophilicity because of OH-filled vacancies, the location of which influences the onset of water adsorption and saturation values.

## 6. Impact of Defects on CNCs/MOFs Functions and Properties

MOFs (*cp*-CNCs) are defined by their regularly repeating crystal structures in which all the pores have exactly the same size, shape, and functionality. This greatly facilitates the establishment of structure–property relationships, which in turn allows us to tailor MOFs for certain targeted applications, such as shape- and size-selective adsorption, catalysis, and sensing. The introduction of defects disrupts the regular porous interior of MOFs and so the behavior for the intended applications can be drastically altered compared to that of the parent MOFs. For example:

1. linker and/or metal vacancies could affect mass-transport pathways within the pores which is important for adsorption and separation processes;
2. metal vacancies might give rise to electronic coupling states, which in turn influence electronic, magnetic, and optical functionalities;
3. defect engineering may prearrange CUSs in a cooperative manner so that complex active sites can be achieved for targeted catalysis within MOFs.

### 6.1. Defect-Engineered CNCs/MOFs in Catalysis

In the past decade, much research has been focused on introducing catalytic species into MOFs, while the role of defects in catalytic processes and the engineering of defect-based active sites in MOFs have only relatively recently gained attention. Almost without exception, the targeted defects for catalysis are coordinative mismatches between the linker and metal ions, which results in Brønsted or Lewis acid sites, respectively. For example, Farrusseng and co-workers synthesized defective MOF-5 and observed that linker vacancies partly occupied by OH groups catalyze the alkylation of biphenyl with *tert*-butyl chloride. While the conversion of biphenyl over fast-precipitated MOF-5 was comparable to that obtained with zeolite H-BEA (28 % versus 30 %), MOF-5 was 100 % *para*-selective, versus only 55 % selectivity for H-BEA.[20], [63] Similar observations were made by Llabres i Xamena et al., who found acidity in MOF-5 as a result of Zn-OH species and free carboxylic acids; this resulted in an active catalyst for the Knoevenagel condensation of benzaldehyde and ethyl cyanoacetate. They further reported on IRMOF-3, which features similar acidic defects, thus making it a bifunctional catalyst by virtue of its structural NH_2_ groups and OH groups of defective origin.[64]

Ameloot et al. exploited the Brønsted acid catalyzed self-condensation of furfuryl alcohol (see Section 5.1). Importantly, the catalytic activity of HKUST-1, MOF-5, and MIL-53(Ga) was found to be directly related to the number of defects.[17] Furthermore, inherent Lewis-acidic defects on Bi^3+^ observed in CAU-7 were shown to be selective catalytic sites in the hydroxymethylation of 2-methylfuran and possessed the right acidity to avoid the acid-induced formation of furan oligomers.[79] Moreover, ZIF-8 was found to catalyze the transesterification of vegetable oils with several aliphatic alcohols by virtue of defect sites at its external surface but not in the bulk micropores. Through combined DFT calculations and CO chemisorption, Chizallet et al. demonstrated that the ratios of acidic Zn^2+^ and NH groups as well as of basic N^−^ and OH groups depend on the operating pressure and temperature of the catalyst.[74] Nevertheless, it is quite likely that other reactions catalyzed by the small-pore ZIFs occur at the external surface and involve reactive defects.[75] Leus et al. reported [VO(BDC)] (MIL-47) to be an active catalyst for the oxidation of cyclohexene. Curiously, according to the literature, such reactions can only proceed over coordinatively unsaturated and accessible V sites. The authors thus stated that such sites (basically defects as defined here) are formed in situ in MIL-47 by the removal of linkers or by partial linker decoordination. A longer induction time for the reaction in *n*-decane than for the reaction in water supports this hypothesis and could point to a partial hydrolysis. However, the presence of inherent V-CUS defects in the as-synthesized materials should not be excluded.[77]

Catalytically active acid sites were also introduced in MIL-100(Fe) by a postsynthetic treatment with a protic acid, which gives rise to an increase in both Lewis and Brønsted acidity because of the protonation and decoordination of a carboxylate group from the Fe_3_O trimers. Consequently, this MOF was first examined as a catalyst in the ene-type cyclization of citronellal to isopulegols.[49] The cyclization of each citronellal isomer results in four isopulegol enantiomers, with the selectivity profile being an indication of the Lewis or Brønsted acidity of the catalyst. The defective MIL-100(Fe) showed a marked increase in selectivity to (−)-isopulegol in comparison to the native material, which is a typical behavior of a Lewis acid. While at first this seems counterintuitive, as the number of Brønsted sites also increases, the authors propose that a cooperative effect between the open Fe site and the COOH group in the immediate vicinity results in a facilitated proton migration, thus favoring formation of the industrially relevant (−)-isopulegol. Furthermore, the acid-treated MIL-100(Fe) was applied as a catalyst in Diels–Alder reactions between 1,3-cyclohexadiene and several dienophiles. Interestingly, it displayed increased activity and selectivity in the reactions with oxygenated dienophiles such as dimethyl fumarate and methyl acrylate, which is attributed to the enhanced activation of the dienophiles at the modified active sites.[49] Another interesting example, concerning postsynthetically generated Lewis-basic sites is the partial removal of nitrate anions as NO_*x*_ species at high temperatures from the Ba-nitrate chains in [Ba(BTC)(NO_3_)] (see Scheme 1).[50] The formed Ba-O^2−^ sites provided a high activity in several Knoevenagel condensation and Michael addition reactions.

Apart from using inherent or engineered Lewis and Brønsted acid sites for catalysis, defect sites can be used to introduce other catalytically active species. For example, Matzger and co-workers showed that up to 2.3 wt % Pd could be grafted on dangling COOH groups in a defect-engineered MOF-5 derivative doped with H_3_BTB.[65] The resulting material appeared to be a good catalyst in the Heck-type phenylation of naphthalene with diphenyliodonium tetrafluoroborate.

**Figure 10:**
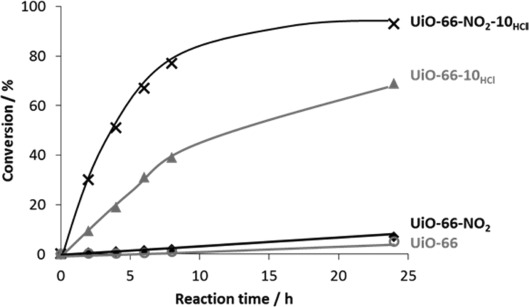
Conversion of TBCH over UiO-66-X and UiO-66-NO_2_-X (X=equivalents of used TFA with respect to other reactants in MOFs synthesis) versus time (toluene, 100 °C, TBCH/IPA/Zr^4+^=10:50:1).[46]

The TML strategy has been used in several materials to modulate catalytic activity. For example, Marx et al. demonstrated a strong increase in the activity of PyDC-doped variants of HKUST-1 compared to the parent MOF in the oxidation of toluene with hydrogen peroxide. Remarkably, the formation of mainly benzaldehyde and *p-*methylbenzoquinone was observed, while the parent HKUST-1 is reasonably selective towards *ortho*- and *para*-cresol.[28] Defect engineering by PyDC doping has also been recently applied to the Ru^2+/3+^-analogue of HKUST-1.[48] In fact, PyDC doping led to materials which feature more strongly reduced Ru^*x*+^ species (*x*<2) as a result of partial reduction of the metal node upon increasing the carboxylate ligator vacancies. Interestingly, those samples demonstrated up to four times higher activity in the hydrogenation of 1-octene, which was attributed to the creation of additional modified Ru-CUSs. Furthermore, De Vos and co-workers attributed the catalytic activity of the UiO-66(Zr) mostly to inherent linker vacancies, which are needed to form realistic transition states.[21] To increase the concentration of linker vacancies in UiO-66, monocarboxylates were used as linker fragments in a follow-up study. A striking example of the efficiency of this approach was the Meerwein–Ponndorf–Verley reduction of 4-*tert*-butylcyclohexanone (TBCH) with isopropanol. While the parent UiO-66 showed almost no activity in this reaction (5 % conversion), the defective MOF was able to convert more than 60 % of TBCH after 24 h (Figure 10).[46]

### 6.2. Defective CNCs/MOFs in Adsorption, Separation, and Storage

In terms of gas adsorption in MOFs, two main properties that can be influenced by defect engineering are the density of the CUS as well as the pore-size distribution and specific surface area. The introduction of vacancy defects in sufficient quantities might result in the formation of mesopores, as explained before. Defect engineering could also be a tool to turn CNCs from a dense, nonporous material (*cd*-CNC) to a porous form (*cp*-CNC), as in the case of a UiO-66 derivative with the small squaric acid (C_4_O_4_H_2_) as linker.[72]

An interesting example of the impact of defects on gas adsorption is NOTT-202, built up from [In(COO)_4_] units linked through biphenyl-3,3′,5,5′-tetra(phenyl-4-carboxylate) linkers and featuring a partially double interpenetrated structure.[36] The first net (A) is fully present whereas the second net (B) is only 75 % occupied and is, moreover, disordered over two positions (B1 and B2) due to symmetry relationships. The resulting framework thus consists of a dominant net A and independent domains of the secondary nets B1 and B2, the occupancy of the latter two each being 37.4 %. The secondary nets B1 and B2, however, cannot be connected to each other because of steric constraints and linker overlap. This induces defect slits with a width defined as the distance between opposite dangling ligands in B1 and B2. The network fragmentation and defects allow the desolvated phase (NOTT-202a) to achieve a high specific surface area (2220 m^2^ g^−1^) and pore volume, despite the interpenetration. The CO_2_ isotherm recorded for this material at 195 K shows three steps in the adsorption profile, thus indicating a stepwise filling of the pores. This behavior is not observed for temperatures above the triple point of CO_2_, which indicates non-ordering of CO_2_ within the pores. Thus, NOTT-202a has a large affinity for CO_2_ and has the potential for its trapping from gas mixtures, as other gases do not show this type of behavior.

**Figure 11:**
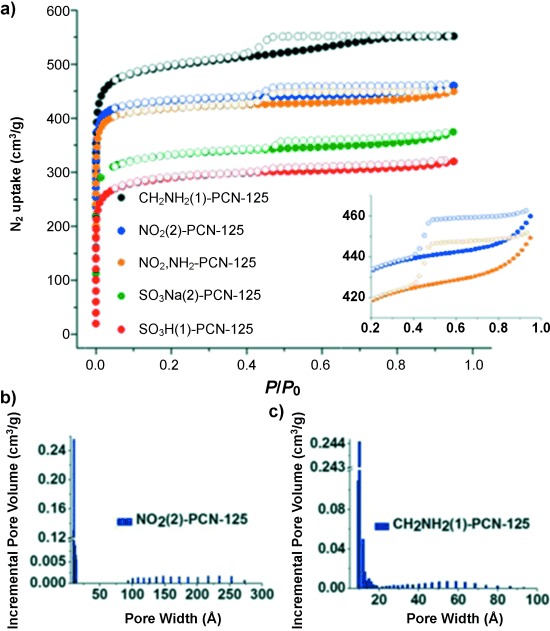
a) N_2_ adsorption isotherms of mesoporous R(*N*)-PCN-125: CH_2_NH_2_(1)-, NO_2_(2)-, NO_2_-, NH_2_-, SO_3_Na(2)-, and SO_3_H(1)-PCN-125. b), c) Pore-size distribution of NO_2_(2)-PCN-125 and CH_2_NH_2_(1)-PCN-125 calculated by DFT.[27]

Wu et al. found that linker vacancies lead to a dramatically enhanced porosity of UiO-66. The pore volume and BET surface area of the samples doped with most linker fragments were found to be 150 % and 60 %, respectively, higher than the theoretical values of the parent material.[19] Furthermore, increased heats of CO_2_ adsorption and mesopore formation were achieved in PCN-125 derivatives containing functionalized linker fragments (Figure 11).[27] MOF-5 synthesized with DBA also features meso- and macropores with a higher CO_2_ adsorption capacity compared to parent MOF-5.[66] Moreover, thermally annealed MOF-5 samples with in situ generated benzoate fragments show higher CO_2_ uptake capacities because of the presence of Zn mCUSs.[69]

Modulation of the porosity by defect engineering was also reported for HKUST-1 and NU-125.[59] Isophthalate-doped derivatives feature increased pore volumes and surface areas, while doping with the more bulky 5-perfluorobutylisophthalate had an opposite effect. The doping with isophthalate derivatives was suggested to cause partial removal of Cu_2_ paddlewheels (ca. 8 %) in HKUST-1. Consequently, because of these metal node vacancies, the total uptake of H_2_ and CH_4_ is reduced despite the higher porosity in some cases. This may be due to the reduced number of adsorption sites involving CUSs. In contrast, an enhancement of MOF performance in sorption-related processes could be reached with defect-generated mCUSs.[48]

Tsao et al. studied the correlations between H_2_ uptake by bridge spillover in MOF-5 and its pore network, specific surface area, and lattice defects. They suggested that aggregated defects, mesopores with a radius ≥ 32 Å, are responsible for variations in H_2_ uptake. Similarly, the occurrence of defect mesopores counteracts pore blocking in IRMOF-8 and increases the uptake capacity for H_2_ up to 4.7 wt %.[62] Such a unique mesopore network combined with other structural defects (i.e. ZnO species in the nanopores and lattice interpenetration as a minor phase) plays an important role in the uptake of H_2_ at room temperature by a bridged spillover mechanism.

### 6.3. Luminescence, Magnetic, Thermal, and Mechanical Properties

As mentioned in the introduction, the defect structure plays a key role in determining the physical properties of solids. MOFs are no exception; however, the influence of defect engineering on their physicochemical characteristics has only been investigated sporadically. Nonetheless, it has been shown that the photoluminescence and magnetic properties of defective CNCs/MOFs differ from their native counterparts. A number of MOFs have received attention because of their photoluminescence properties and have been recently reviewed.[113]–[115] It is well known that defects play a considerable role in the optical properties of diverse solid-state materials.[114] For example, defect-induced luminescence has been found in Co^2+^-doped anatase, Cd-rich CdS and ZnS nanoparticles, and Eu^2+^-doped Sr_2_MgSi_2_O_7_. Defects may act either as energy-storage centers, which introduces luminescence, or as quenching centers in materials. For example, defects in Sr_2_MgSi_2_O_7_ act as electron traps close to the material’s conduction band. These sites store absorbed energy and contribute to an efficient persistent luminescence.[116] Larsen and co-workers reported that [Ru(2,2′-bipyridine)_3_]^2+^, encapsulated in volume defects in RWLC-2 (built from H_3_BTB and Zn^2+^ ions), features reduced emission lifetimes compared to in the solution state because the co-confinement with a quenching molecule in defect voids activates altered decay channels.[83]

Defects could also allow modification of the electronic band structure of CNCs. In fact, larger band gaps ranging from 3.31 to 3.87 eV were found in TML-modified HKUST-1, while a value of 2.87 eV was reported for the hydrated parent HKUST-1.[24] This is associated with both the doped amount of linker fragments and the concomitant partial reduction of Cu^2+^ to Cu^+^.

Structural defects might also alter phonon transport, thereby affecting the thermal conductivity of solids. For example, this was shown for β-SiC, in which phonon scattering by defects strongly reduces the thermal conductivity.[117] Kaviany and co-workers reported the dependence of the thermal conductivity of MOF-5 on the mode of phonon scattering. The phonon mean free path length in MOF-5 reaches a minimum below 13 K, which is typically attributed to point defects (either inherent or formed through thermal stresses). As revealed, thermal conductivity increases with increasing temperature, and scattering by lattice defects is an important phonon scattering mechanism below 35 K.[67], [68]

The deliberate introduction of metal ion defects, for example, forming mixed-metal MOFs, could open a promising door to modifying the magnetic properties of CNCs. For example, Williams and co-workers observed a ferromagnetic coupling between Cu_2_ paddlewheels in HKUST-1, with a dramatic rise in the magnetic susceptibility below 70 K.[118] Motivated by vacancy-induced magnetism in many materials, Shen et al. further proposed the ferromagnetism in a series of Cu_2_ paddlewheel based MOFs to be induced by vacancy defects.[80] Their DFT results excluded effects of other possible defects (such as atomic C and O vacancies) on the induction of any magnetic moment because of the delocalization of their defect wave functions. Both theoretical and experimental data indicated that the observed ferromagnetism is due to point defects, that is, Cu^2+^ vacancies. A clear correlation between magnetic properties and the doping level of PyDC was found in TML-engineered HKUST-1.[24]

Finally, the impact of defects on the mechanical properties of MOFs was reported by Goodwin and co-workers, who used ab initio calculations to predict the mechanical stiffness of UiO-66(Hf) containing the correlated **reo** defect domains. Their calculations revealed a strong decrease in stiffness (Young’s modulus for parent UiO-66 equals 46.8 GPa, for the defective **reo** regions 23.3 GPa), which potentially could lead to pressure-induced amorphization.[23] Defect-induced anomalous mechanical properties might be a promising field that could draw future interest.[119]

## 7. Conclusions and Further Directions

Defect structure characterization and defect engineering have been mainly connected with catalysis and gas sorption. This area could mature in the coming years, especially benefiting from deliberately induced linker and metal ion node vacancies. In this sense, many MOFs whose parent structure does not allow much diversity (e.g. steric and electronic restrictions at the framework metal ion sites) can be considered as “sleeping beauties”, in which active sites can be unlocked by careful engineering of defects. We expect that the lower coordination number of defect metal ion sites will greatly affect the selective sorption properties of CNC materials with respect to gas separation and storage and also their catalytic activity because of the modified steric and electronic environments in the material. Defect engineering will lead to materials with a multivariate nature, which could be interesting for the implementation of multiple functionalities in these materials. In general, we propose that the investigation of the (external) surface defect structure of essentially nonporous *c*-CNCs may also be important for the application of such materials in heterogeneous catalysis. It is also clear that using MOFs (*cp*-CNCs) for catalysis and also for biomedical applications (e.g. drug carriers, drug targeting, cell function control) does actually require some scaling down of the crystallites to the nanosize regime and/or the implementation of hierarchical porosity (macro-meso-micro), because diffusion limitations need to be taken into account. The synthetic methods of downscaling themselves, however, are intimately connected with the formation of various kinds of defects and structural heterogeneity, as we have pointed out. Thus, the connection between all these issues needs to be considered in the future.

### 7.1. Synthesis, Characterization, and Modeling

The intentional implementation of defects in CNCs/MOFs on various length scales, thereby causing structural and compositional complexity, is beginning to emerge as a part of the research efforts to develop new classes of functional molecular network materials. While the role of defects in other solid-state materials such as oxides has been well-established, the pronounced effects of defects on the properties of CNCs/MOFs deserve much more attention. The very nature of coordination network compounds, in general, being crystalline scaffolds of inorganic metal ion nodes connected by organic linkers, offers great opportunities for defect engineering. The relative ease by which multiple components such as fragmented linkers and redox active metal ions can be introduced into metal–organic frameworks is a prime example of this. Nevertheless, there is a clear need to investigate the full range of possible defect types and new methods by which they can be introduced into CNCs/MOFs with some control. For example, the formation of defects by irradiation with high-energy ions, which has been used to create mesopore defects in zeolites,[120] could be equally efficient in CNCs/MOFs. Another important aspect is the application and the development of characterization techniques to unambiguously establish the nature and distribution and/or correlation of defects, as only from this information will it be possible to rationally deduce engineering strategies. We highlighted some of the currently employed experimental characterization methods, including chemisorption combined with spectroscopic techniques, as is the case for FTIR with a CO probe, gas adsorption techniques to detect changes and anomalies in the nature of the pore system, and several imaging techniques such as SEM and AFM to study surface defects. More advanced techniques can provide a lot of useful information concerning defects, for example the use of pair distribution function (PDF) analysis of X-ray or neutron diffraction data, or CFM to directly image dislocations within a material. Scanning tunneling microscopy (STM) as a high-resolution technique is ideally suited to study structural properties of surface-confined 0-, 1-, and 2D molecular arrangements in real space not only of pristine metal and semiconductor surfaces, but also of adsorbate-covered surfaces.[121] This technique might be suitable for the study of various surface defects of electrically conductive MOF/CNC thin films. Such samples should of course allow a current flow between the probe tip and the sample.

Standard techniques for structure determination such as diffraction methods are insensitive to local defects, whereas spectroscopic investigations do not provide direct structural information. Thus, for an in-depth understanding of defect engineering, clearly, theoretical methods are needed as an additional tool to predict the relative stability of potential defective structures and to support the interpretation of spectroscopic results. Up to now, only a few research groups have combined comprehensive experimental methods and calculations for this purpose. Future directions of research will take into account that the approximation of periodic boundary conditions cannot easily be maintained for the modeling of dilute defects embedded in a regular crystalline environment. To accurately capture this situation within a high-level and full periodic quantum mechanical DFT calculation, a large unit cell must be used, thus leading to a very large, if not unmanageable numerical effort. On the other hand, because of the molecular nature of the investigated systems, electronic interactions (correlations, also of defects) are very local. Thus, specific multiscale simulation techniques for defective CNCs/MOFs need to be developed. Based on a novel genetic algorithm optimization strategy, an accurate force-field parametrization can be derived in a systematic way from first principles reference data of nonperiodic model systems. It was shown that complex dynamic effects such as negative thermal expansion could be quantitatively predicted by such a method.[122] Furthermore, the controlled introduction of defects during the synthesis of CNCs/MOFs is directly connected to the growth mechanism of the crystalline materials. Essentially, this refers to the chemistry of the (external) solid/liquid interface. Only recently has computational modeling of the external surface structure of CNCs/MOFs (surface termination) been done, again with HKUST-1 as the prototypical case.[123] This study is immediately connected with the coordination modulation concept and, therefore, points towards the mechanisms of fragmented linker incorporation as well. For example, the missing-node defect type created by the TML approach can be regarded as creating an internal surface termination. At this point we want to connect the defect topic with the solvent-assisted linker exchange (SALE) concept.[124]–[129] SALE has already been demonstrated as an important strategy for the generation of a variety of multifunctional MOF materials previously unobtainable by direct synthesis methods. It was recently reviewed by Hupp, Farha, and co-workers.[129] Herein, we propose that defects with missing or weaker coordination bonds in the framework will facilitate SALE. Furthermore, the mechanism of SALE may well be connected to the defect structure and/or dynamic defects of the parent frameworks. Consequently, the engineering of defects and its theoretical modeling may well be relevant to a better understanding of the mechanism of SALE.

### 7.2. Defect Structure Related Properties and Functions

In general, the physical properties of CNCs/MOFs and novel functions related to the “physics” of the materials need to be investigated with much more emphasis than in the past. If so, then understanding the defect structure will become even more important. For example, metal ions and counterions could be grafted at defects to control the ionic conductivity. The resulting solid electrolytes will be potentially useful for enhancing the operation of next-generation lithium batteries.[130] Starting from the pioneering work of Shen et al.[80] on metal-ion vacancies inducing a long-range ferromagnetic ordering, it will be promising to fabricate new ferromagnetic materials by designing CNCs/MOFs with deliberate metal-ion vacancies. Furthermore, such defects could act as energy-storage sites that promote persistent luminescence. The incorporation of defects further offers the possibility to fine-tune band gaps for optical applications. A breakthrough in CNC/MOF materials research would certainly be the realization of the combination of electrical conductivity with tunable porosity and the design of the internal coordination space. Such kinds of materials would combine features known from organic semiconductors and “organic synthetic metals” with the reticular chemistry of MOFs. If such materials are developed it is immediately evident that understanding and controlling the respective defect structure for band-gap engineering, the charge-carrier concentration, and mobility will be as important as for other electronically conductive materials. While a huge amount of knowledge exists on conductive (nonporous) coordination polymers, an explicit and systematic investigation of the control of the conductive properties of defect structures has only been done within the context of doping with redox-active components, which is somewhat different from the viewpoint of our discussion.

We want to draw attention to two recent and important results which point towards the more realistic possibility to achieve materials for “MOFtronics”.[131] HKUST-1 was turned into an Ohmic conductor by loading with TCNQ (TCNQ=tetracyanochinodimethane).[132] Coordination of TCNQ at the native Cu-CUSs led to a drastic increase in the conductivity to reach optimum values of 7×10^−2^ S⋅cm^−1^, however, simultaneously compromising the porosity (drop from the parent 1840 to 210 m^2^ g^−1^ for the loaded material). A more detailed investigation of the local, short-range, and the longer-range defect structure (grain boundaries) would certainly be important to better understand the origin and details of charge-transport mechanisms. Tailoring the porosity by the TML approach, similar to the work of Barin et al.[59] mentioned above, would add another dimension of engineering to the material properties. Furthermore, we want to mention the new semiconductive, graphene-like porous material [Ni_3_(HITP)_2_]_n_ (HITP=2,3,6,7,10,11-hexa-iminotriphenylene) with a thin-film conductivity of 40 S⋅cm^−1^.[133] The strictly regular, crystalline nature of the parent material frameworks, in principle, allows more rigorous theoretical investigations and searching for the compositional and structural conditions needed for the development and identification of promising candidates for “MOFtronics”.[131]

## Abbreviations

AFM: atomic force microscopy
BDC: 1,4-benzenedicarboxylate
BET: Brunauer–Emmet–Teller
BPP: 1,3-bis(4-pyridyl)propane
bpy: 2,2′-bipyridine
BTC: 1,3,5-benzenetricarboxylate
BTB: 1,3,5-benzenetrisbenzoate
CAU: Christian–Albrechts University
CFM: confocal fluorescence microscopy
CNC: coordination network compound
*c*-CNC: crystalline CNC
*a*-CNC: amorphous CNC
*r*-CNC: rigid CNC
*f*-CNC: flexible CNC
*p*-CNC: porous CNC
*d*-CNC: dense CNC
CP: cross-polarization
CUS: coordinatively unsatured site
mCUS: modified CUS
DBA: 4-(dodecyloxy)benzoic acid
DFT: density functional theory
DLS: dynamic light scattering
DR: diffuse reflectance
DRIFTS: DR infrared Fourier transform spectroscopy
DSC: differential scanning calorimetry
EDX: energy dispersive X-ray spectroscopy
EPR: electron paramagnetic resonance
EXAFS: extended X-ray absorption fine structure
FMM: force modulation microscopy
FTIR: Fourier transform infrared
H_2_BDC: 1,4-benzenedicarboxylic acid
H_3_BTB: 1,3,5-benzenetrisbenzoic acid
HITP: 2,3,6,7,10,11-hexaiminotriphenylene
HKUST: Hong Kong University of Science and Technology
HML: heterostructural mixed linker
HPLC: high-performance liquid chromatography
IML: isostructural mixed linker
IPA: 2-propanol (“isopropanol”)
IRMOF: isoreticular MOF
LML: large mixed linker
MAS-NMR: magic-angle spinning NMR
MIL: material of the Institut Lavoisier
MOF: metal–organic framework
NDC: 2,6-naphthalenedicarboxylate
NOTT: Nottingham University
NU: Northwestern University
P_3_HT: poly(3-hexylthiophene)
PALS: positron annihilation lifetime spectroscopy
PCN: porous coordination network
PDF: pair distribution function
pmg*-*: “pomegranate”-like MOF
PXRD: powder X-ray diffraction
PyC: 4-pyrazolecarboxylate
PyDC: 2,5-pyridinedicarboxylate
QM/MM: quantum mechanics/molecular mechanics
SALE: solvent-assisted linker exchange
SAM: self-assembled monolayer
SBU: secondary building unit
SEM: scanning electron microscopy
spng: “sponge”-like MOF
STM: scanning tunneling microscopy
SURMOF: surface-mounted MOF
TBCH: 4-*tert*-butylcyclohexanone
TCNQ: tetracyanochinodimethane
TEM: transmission electron microscopy
TFA: trifluoroacetic acid
TGA: thermogravimetric analysis
TML: truncated mixed linker
UiO: Universitetet i Oslo
UHV: ultrahigh vacuum
XANES: X-ray absorption near-edge structure
XPS: X-ray photoelectron spectroscopy
ZIF: zeolitic imidazolate framework
